# Leveraging MedlinePlus to Improve Health Information Access Among Patients and Caregivers: Systematic Literature Review

**DOI:** 10.2196/79416

**Published:** 2026-04-27

**Authors:** Fei Yu, Xiaomeng Wang, Jia Liu, Tian Wang, Siobahn Day Grady, Lixin Song

**Affiliations:** 1 Carolina Health Informatics Program, School of Information and Library Science, University of North Carolina at Chapel Hill, 216 Lenoir Drive CB #3360, 100 Manning Hall, Chapel Hill, NC, 27599, United States, 1 919663589; 2 McWilliams School of Biomedical Informatics, University of Texas Health Science Center at Houston, Houston, TX, United States; 3 University of Texas Health Science Center at San Antonio, San Antonio, TX, United States; 4 School of Information Sciences, University of Illinois at Urbana‑Champaign, Champaign, IL, United States; 5 School of Library and Information Sciences, North Carolina Central University, Durham, NC, United States

**Keywords:** MedlinePlus, health information access, information intervention, information prescription, patients and caregivers, systematic literature review

## Abstract

**Background:**

MedlinePlus, developed by the National Library of Medicine (NLM) in the United States, is one of the most widely used, authoritative, consumer-grade health information resources on the web. Although extensively used and discussed in scholarly work for health literacy and patient education, it is unclear how MedlinePlus has been integrated into clinical care or embedded within health informatics applications.

**Objective:**

This study aimed to understand how MedlinePlus has supported patients and caregivers by increasing access to health information for clinical care and illness management. The insights on this topic will inform the design and development of patient-facing digital health intervention tools for improved health communication, decision engagement, informed decision-making, and health outcomes.

**Methods:**

We conducted a systematic literature review following the PRISMA (Preferred Reporting Items for Systematic Reviews and Meta-Analyses) guidelines. First, we developed a comprehensive literature search strategy, searched 9 citation databases, and aggregated and deduplicated search results before importing them into Covidence for manual screening using predefined inclusion and exclusion criteria. Second, reviewers independently assessed all studies at the title-abstract and full-text levels, resolving discrepancies through ongoing discussions. Third, we applied the PICO (problem/population, intervention, comparison, and outcome) and the Collaborative Chronic Care Model as guiding frameworks for data extraction and analysis. All included studies underwent quality assessment using the Mixed Methods Appraisal Tool.

**Results:**

In total, 28 studies reported in 27 sources met our inclusion criteria. We categorized the extracted data into 4 areas. First, regarding bibliometrics, the studies were reported between 2004 and 2024, with 2010 having the highest number of studies. Of these studies, 25 were conducted in the United States, 2 were conducted in Iran, and 1 was conducted in Argentina. Health informatics journals and conference proceedings, as well as library science journals, were prominent publishing venues. The NLM funded half of the studies. Second, regarding participants, most studies focused on outpatients. Other participant roles included physicians, nurses, hospital staff, pharmacists, and librarians. Fewer than half of the studies addressed the social determinants of health. Third, regarding intervention, most studies implemented MedlinePlus information interventions within clinical settings. Other interventions occurred in community pharmacies, community organizations, libraries, online health platforms, or patient portals. Fourth, regarding outcome, only 4 studies assessed clinical outcomes, and the findings were mixed and inconsistent. However, 24 of 28 studies reported positive nonclinical outcomes, including improved attitudes toward and satisfaction with MedlinePlus and enhancements in patients’ information-seeking behaviors, confidence, and willingness to engage in decision-making, physician-patient communication, self-management, and self-efficacy.

**Conclusions:**

This systematic literature review is the first comprehensive examination of how MedlinePlus has been integrated into clinical care, supporting patients and caregivers with enhanced access to health information. Our findings offer evidence and insights through the Collaborative Chronic Care Model lens and can guide the development of digital health interventions to improve patient health.

## Introduction

The National Library of Medicine (NLM) in the United States launched the MedlinePlus website in 1998 to provide the public with free access to “high-quality, relevant health and wellness information that is trusted, easy to understand, and free of advertising” [1]. Being available in both English and Spanish, MedlinePlus provides content covering more than 1000 diseases and health conditions, almost 300 medical test descriptions, more than 1500 brand-name and generic drugs, 1300 genetic conditions and 1400 genes, and dietary supplements and herbs, as well as more than 4000 medical encyclopedia articles and healthy recipes. In addition to textual content, MedlinePlus offers animated health videos produced by the NLM or selected from accredited external organizations and plentiful interactive health assessment tools, enabling people to assess their risk for diseases or health conditions and maintain healthy lifestyles [2].

Over the past two decades, the NLM has continuously expanded and enhanced MedlinePlus by adding new features and content to meet the evolving needs of its users. In particular, in the era of electronic health records (EHRs), when patients’ and caregivers’ engagement in shared decision-making is empowered by their access to medical records, the NLM activated MedlinePlus Connect [3]. This application programming interface service links MedlinePlus content to patient portals and EHRs, greatly extending the scope of MedlinePlus information availability, compatibility, and usage. For example, studies show that patient portals embed MedlinePlus to improve medical record comprehension [4,5].

MedlinePlus has become a widely used free health information resource, serving millions of users worldwide and supporting health care professionals, educators, and researchers in providing evidence-based health education and improving health literacy. In particular, it has been frequently cited in the scholarly literature on the concept and practice of information prescription (IP) because of its demonstrated utility in patient care. IP refers to the physician’s prescription of current, personalized, evidence-based, and reliable health information to patients as part of their treatment plan to understand, manage, and control their health problems and to better engage in their health care [6-8]. Although IP can take paper or electronic format, the Information Rx (IRx) program, initiated in 2002 by the American College of Physicians Foundations and the NLM, provided physicians with prescription pads designed to guide patients to relevant content on the MedlinePlus website, tailored to their specific health conditions [9]. The IP and IRx initiatives positioned MedlinePlus as one of the key resources in disseminating consumer-grade health information. A recent scoping review confirmed that “the most commonly prescribed website to produce IP content is MedlinePlus,” compared with 15 other identified IP contents [8].

Although researchers unanimously agree on the value of IP and IRx in patient education, self-management, and decision engagement, the integration of MedlinePlus into clinical practice, particularly embedding it within health care applications such as EHRs or digital health interventions, is unknown. No studies have systematically examined the extant research evidence about how MedlinePlus has been adopted, implemented, or integrated to improve patient care and health outcomes, especially in the context of designing and developing health information technology (Health IT). To address this gap, this study aimed to investigate the role of MedlinePlus in supporting patients and their caregivers by increasing access to health information pertinent to their care and illness management, with a focus on Health IT. A systematic mapping of the literature will inform the design and development of patient-centered digital health interventions that enhance health communication, decision engagement, informed decision-making, and health outcomes.

## Methods

This study used a systematic literature review methodology, adhering to the PRISMA (Preferred Reporting Items for Systematic Reviews and Meta-Analyses) guidelines (Checklist 1) [10] for literature search, study selection, and data extraction.

### Literature Search

A literature search strategy was first developed by connecting the 2 main keywords “MedlinePlus” and “Medline Plus” with the Boolean operator “OR” and then customizing the query based on the field codes of the chosen citation database. This strategy was executed across 9 citation databases, including PubMed, Embase, CINAHL, Global Health, PsycINFO, Cochrane Database of Systematic Reviews, Scopus, Web of Science, and Dimensions. The first 5 databases are well-known research article indexing resources in the health sciences and are primary sources for clinical studies, including Health IT and digital health interventions. We also searched the Cochrane reviews, the gold standard for systematic reviews in biomedical and health sciences, to identify relevant reviews and examine their cited empirical studies, ensuring comprehensive coverage of the literature. Furthermore, Scopus, Web of Science, and Dimensions were included because they index a broad range of peer-reviewed research articles across medicine and health sciences and are commonly used in systematic reviews and evidence-mapping studies. Our initial search was conducted in October 2021 and was updated in October 2022 and in September 2024 (Multimedia Appendix 1). In addition, we checked all references of the included articles (backward searching) and newer articles that cited the included studies (forward searching) in Scopus for an exhaustive literature search.

### Study Selection

The search results from each database were exported, aggregated, deduplicated, and imported into Covidence [11] for screening. Independent reviewers from our research team conducted the screening process, initially at the title-abstract level (FY, Erin Harvey, and Pengxuan Wang), followed by a full-text assessment (FY and XW). Discrepancies between reviewers were resolved through discussion to achieve agreement.

The studies were included if they (1) used MedlinePlus as the primary or one of the primary information or education resources for patients and/or their caregivers; (2) targeted patients or caregivers, as indicated by the recruited participants or by the research aims; (3) were English literature; and (4) were original research articles. The studies were excluded if they were (1) non-English literature, (2) did not use MedlinePlus as one of the information sources, (3) did not include patients or caregivers as direct information recipients (eg, only physicians or other health care professionals are information or training recipients), (4) were not original research articles (eg, perspectives, comments, editorials, news, bulletins, erratum, or abstract only), (5) were information readability or coverage assessment studies, or (6) were information retrieval, search behavior, or query analysis studies.

### Data Extraction and Synthesis

To achieve the research aim, we adopted 2 frameworks to guide our data extraction and synthesis process. The first is the PICO (problem/population, intervention, comparison, and outcome) framework [12], widely recognized and used in evidence-based practice and research in health sciences. The PICO framework systematically addresses the targeted population, specific intervention or exposure, intervention comparison to an alternative (if applicable), and desired outcomes or end points. The second framework is the Collaborative Chronic Care Model (CCCM) [13], a refined version of the widely adopted original chronic care model. The CCCM emphasizes collaborative, continuous, evidence-based, and proactive care delivery for individuals with chronic, medical, or mental health conditions. The CCCM encompasses 6 major elements: health system organization, work role redesign, self-management support, decision support, information management, and community linkages. We created a customized data extraction framework (Table 1) and organized the data accordingly. Guided by the PICO and CCCM frameworks, our data extraction focused on the following 4 aspects: bibliometrics, participants, interventions, and outcomes.

**Table 1.** Data collection, extraction, and analysis framework.

Measure	Data extraction metrics	Data source	Analysis
Bibliometrics	Study distribution by year, source, country, and funding; authors and institutions; and citation and digital impact	Full-text article, Scopus, Altmetric Explorer	Quantitative
Participants	Participants’ role, diagnosis, demographics (including SDOH^a^), sample size, and age range	Full-text article	Qualitative and quantitative
Interventions	Intervention features, information content, additional interventions, and setting	Full-text article	Qualitative
Outcomes	Clinical outcome, nonclinical outcome, outcome comparison, study duration, and follow-up method	Full-text article	Qualitative and quantitative

a SDOH: social determinants of health.

### Bibliometrics

Bibliometrics capture the document details of the included studies, including reporting year, source title, funding sources, author names and affiliations, and citation impact. We assessed the citation impact of included studies using both raw citation counts (ie, how many times an article has been cited) and the field-weighted citation impact (FWCI), a field- and time-normalized citation impact metric [14] provided by the Elsevier Scopus database. An FWCI of 1 indicates that, compared with papers published in the same field and time, this article is cited at the global average level; a value greater than 1 suggests that it is cited more than the worldwide average and vice versa. Being an improved citation impact measure, FWCIs have been commonly adopted by research impact evaluators [15,16]. In addition, we used Altmetrics to capture the publication’s digital attention [17], with scores obtained from Altmetric Explorer [18] through an institutional subscription. The composite Altmetric Attention Score (AAS) is a weighted and quantified measure of the online attention and engagement that a research output (eg, journal article) has received by tracking various sources such as public policy documents, mainstream news, social networks, blogs, and other platforms. Generally, an AAS of 20 or higher is considered good [19] and is often used as a benchmark in research evaluations [20].

### Participants

The participant category captures participants’ roles, patient diagnoses, CCCM-aligned demographics (including social determinants of health [SDOH]), sample size, and age range of patients and caregivers who used MedlinePlus. Participant roles include physicians, nurses, research associates, patients, and caregivers involved in using MedlinePlus as an information intervention. Demographics include SDOH, such as self-management skills, education and literacy levels, and economic and financial factors (eg, income, employment, and health insurance). Reporting SDOH and demographic characteristics (eg, race, gender, and primary language) provides contextual information and aligns with CCCM's emphasis on whole-person care and linkage to community resources.

### Interventions

Interventions describe how MedlinePlus was used as a health information intervention, including the type of content assessed, platform features used, and modes of delivery. Using the CCCM-aligned construct of intervention delivery, we systematically documented the key intervention attributes, including intervention features, health information content, any additional interventions beyond the MedlinePlus resource, and the settings where the interventions were implemented.

### Outcomes

Outcomes cover clinical and nonclinical outcomes as well as outcome comparison and assessment, if applicable. Clinical outcomes are associated with “a measurable change in symptoms, overall health, ability to function, quality of life, or survival outcomes that result from giving care to patients” [21]. Nonclinical outcomes encompass a wide range of measures, including participants’ attitudes toward health information resources, health information–seeking behavior, experience with health information interventions, information usage, and perceptions. We also examined the study durations and follow-up methods used to assess outcomes of MedlinePlus as an information intervention.

In total, 3 reviewers (FY, Pengxuan Wang, and XW) independently extracted data from the Scopus database, Altmetric Explorer, and the full-text articles of the included studies. We recorded data in a Microsoft Excel template for each included study based on our data extraction framework and discussed and reconciled data inconsistencies to reach consensus.

### Quality Assessment

Two reviewers (FY and XW) independently assessed the quality of the included studies using the Mixed Methods Appraisal Tool (MMAT) [22] by following the MMAT user guide. MMAT has been widely used in systematic reviews to appraise qualitative studies, quantitative studies, randomized controlled trials (RCTs), nonrandomized studies, and mixed methods studies [23,24]. The MMAT tool offers a checklist. First, each study undergoes 2 screening questions to ensure that it is an empirical study (ie, “S1. Are there clear research questions?” and “S2. Do the collected data allow addressing the research questions?”). Second, reviewers select an appropriate study category for each included study. Third, based on the 5 criteria in the selected category, reviewers rate each criterion as “Yes,” “No,” or “Can’t tell,” documenting any observations in a “Comments” column. We rated “Yes” when a study provided sufficient information to fully comply with the criteria, “No” when the study failed to meet the criteria, and “Can’t tell” when the information in the full-text article was insufficient, unclear, or partially met the criteria.

After discussing and resolving discrepancies between the 2 reviewers’ independent ratings, the research team achieved consensus and presented the final rating results at the criteria level for all included studies, following the MMAT user guide (Multimedia Appendix 2).

## Results

### Literature Search and Study Selection

**Figure 1.**
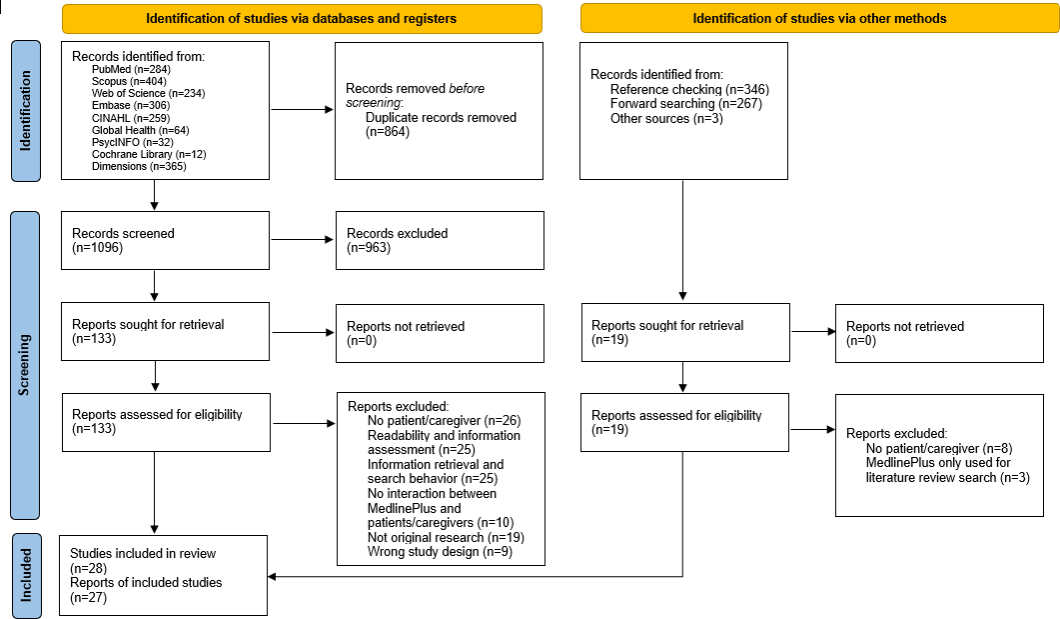
PRISMA (Preferred Reporting Items for Systematic Reviews and Meta-Analyses) flow diagram of the systematic literature search, screening, and study selection process [10].

In total, 28 studies reported in 27 sources met our inclusion criteria (Figure 1). Cohen κ measured the interreviewer agreement, which was 0.63 at the title-abstract level and 0.9 at the full-text level, indicating substantial agreement between reviewers. The results of all included studies are delineated by 4 categories of measures in Tables 2-5.

**Table 2.** Bibliometric characteristics of included studies.

Study	Source title	Country	Funding source	Citation count, n	FWCI^m^	AAS^n^
D'Alessandro et al, 2004 [25]	Archives of Pediatrics & Adolescent Medicine	USA	RWJ^c^	77	2.45	7
Siegel et al, 2006 [9]	Information Services & Use	USA	N/A^d^	43	2.61	N/A
Hailemeskel et al, 2007 [26]	Journal of the American Pharmacists Association	USA	NLM^e^	1	N/A	N/A
Leisey and Shipman, 2007 [27]	Journal of the Medical Library Association	USA	NLM	20	0.87	2
Smalligan et al, 2008 [28]	Journal of Investigative Medicine	USA	N/A	11	0.46	N/A
Zyskind et al, 2009 [29]	Information Services and Use	USA	NLM	7	N/A	N/A
McConnaughy and Wilson, 2010 [30]	Journal of Consumer Health on the Internet	USA	NNLM^f^	N/A	N/A	N/A
Coberly et al, 2010 [6]	Journal of the Medical Library Association	USA	NLM	25	1.84	3
Teolis et al, 2010 [31]	Journal of Consumer Health on the Internet	USA	NLM	15	0.5	3
Ulmer and Robishaw, 2010 [32]	Journal of Consumer Health on the Internet	USA	N/A	10	N/A	N/A
Lasky et al, 2011 [33]	Journal of Consumer Health on the Internet	USA	N/A	2	0.17	N/A
Coberly et al, 2012 [7]	Informatics in Primary Care	USA	NLM	6	0.6	1
Gavgani, 2012 [34]	Book Chapter in Evidence-Based Medicine	Iran	N/A	N/A	N/A	N/A
McCarthy et al, 2013 [35]	Annals of Emergency Medicine	USA	NLM and AHRQ^g^	21	1.73	63
Tarver et al, 2013 [36]	Medical Reference Services Quarterly	USA	NLM or NNLM	6	0.18	N/A
Ramesh et al, 2013 [37]	Studies in Health Technology and Informatics	USA	NCATS^h^	40	5.79	N/A
Borbolla et al, 2014 [4]	Studies in Health Technology and Informatics	Argentina	N/A	17	3.36	1
Koonce et al, 2015 [38]	Journal of the Medical Library Association	USA	IMLS^i^	20	1.44	1
Ancker et al, 2016 [5]	AMIA Annual Symposium proceedings	USA	AHRQ and NLM	15	0.95	5
Wilcox et al, 2016 [39]	Journal of the American Medical Informatics Association	USA	AHRQ, NLM, and NINR^j^	33	1.9	9
Caufield-Noll and Gorman, 2017 [40]	Journal of Consumer Health on the Internet	USA	NLM	2	0.32	N/A
Sanders et al, 2018 [41]	Journal of the Medical Library Association	USA	NLM	2	0.11	1
Fenske, 2019 [42]	Journal of Hospital Librarianship	USA	NLM or NNLM	1	0.17	N/A
Fawcett, 2020 [43]	Dissertation	USA	N/A	N/A	N/A	N/A
Kazemi Majd et al, 2021 [44]	Health Informatics Journal	Iran	Tabriz University of Medical Sciences	5	0.33	5
Zhang et al, 2023 [45]	Journal of the American Medical Informatics Association	USA	NLM and NCATS	N/A	N/A	1
Yu et al, 2024 [46]	IEEE 12th International Conference on Healthcare Informatics	USA	DoD^k^	N/A	N/A	N/A

a FWCI: field-weighted citation impact.

b AAS: Altmetric Attention Score

c RWJ: Robert Wood Johnson Generalist Faculty Scholars Program.

d N/A: not reported in the full-text article.

e NLM: National Library of Medicine.

f NNLM: National Network of Libraries of Medicine.

g AHRQ: Agency for Healthcare Research and Quality.

h NCATS: National Center for Advancing Translational Sciences.

i IMLS: Institute of Museum and Library Services.

j NINR: US National Institute of Nursing Research.

k DoD: (US) Department of Defense.

### Bibliometrics

**Figure 2.**
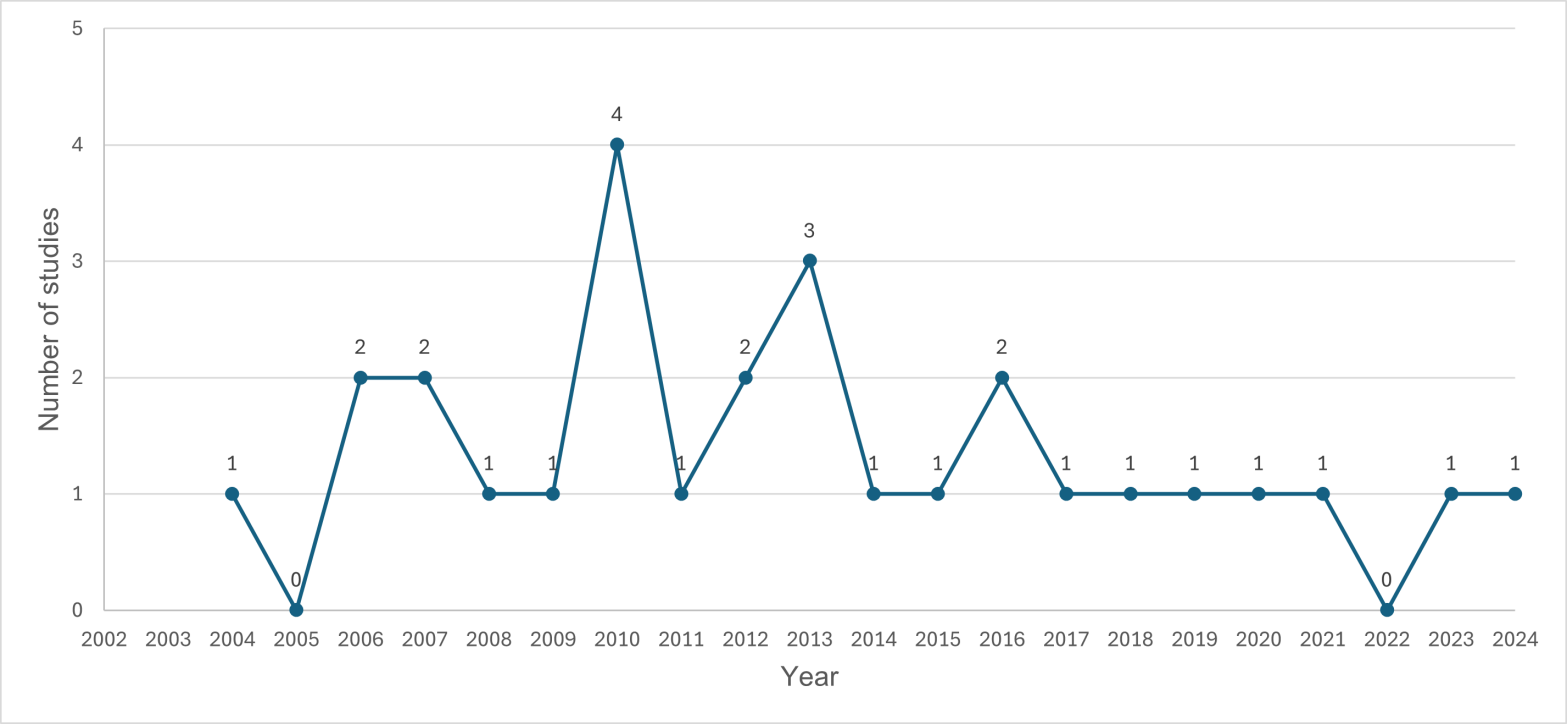
Number of included studies by year (2004-2024).

The included studies were reported between 2004 and 2024 (Table 2). The year 2010 had the highest number of studies (n=4), followed by 2013 (n=3). The years 2006, 2007, 2012, and 2016 each had 2 studies, while all other years had either 1 or no studies (Figure 2).

Of the 28 studies, 25 were conducted in the United States, with 2 conducted in Iran [34,44] and 1 conducted in Argentina [4]. The document types varied and included 1 book chapter [34], 4 health informatics conference proceedings (ie, AMIA Annual Symposium [5], Studies in Health Technology & Informatics [4,37], and IEEE International Conference on Healthcare Informatics [46]), and 1 doctoral dissertation [43]. The remaining studies were published in 12 academic journals. The *Journal of Consumer Health on the Internet* was the most frequent publication venue, with 5 publications, followed by the *Journal of the Medical Library Association* (4 publications), the *Journal of the American Medical Informatics Association* (2 publications), and *Information Services & Use* (2 publications).

In total, 20 publications reported funding support from 9 agencies. The NLM was the primary funder, supporting 15 studies. Other funders included several US federal agencies (eg, Agency for Healthcare Research and Quality, National Center for Advancing Translational Sciences, National Institute of Nursing Research, and Department of Defense), 1 foundation program (ie, Robert Wood Johnson Generalist Faculty Scholars Program), and Tabriz University of Medical Sciences in Iran.

A total of 125 individuals from 65 organizations contributed to the included studies. Notable contributors included authors from the NLM (3 publications), George Washington University (2 publications), University of Missouri (2 publications), and Columbia University Medical Center (2 publications).

Of the 27 sources, 22 were cited in scholarly works. Eight publications had FWCI scores above 1.0, with the study by Ramesh et al [37] having the highest FWCI of 5.79 (Table 2), indicating that the publication has received more citations than expected for its field. Altmetric scores were available for 11 publications, with the highest score of 63 by McCarthy et al [35], indicating that it is widely shared, discussed, and referenced across multiple platforms. This paper is also the only one with an AAS above 20, indicating high engagement (frequent mentions across multiple sources, including news, policy reports, and mainstream discussions).

### Participants

Of 28 studies, 18 involved outpatients, while 5 focused on inpatients and their family members. Notably, 2 studies designed digital information interventions incorporating MedlinePlus but recruited their participants for testing either through crowdsourcing [37] or from a local community [45]. Patients represented a range of clinical conditions, such as diabetes, cardiovascular or heart valve diseases, pediatric cancer, heart failure, and depression. More than half of the studies recruited patients with multiple or varied diagnoses (Table 3).

**Table 3.** Participant characteristics of included studies.

Study	Participants’ role	Patient diagnosis	Demographics and SDOH^a^	Sample size, n	Age range (y)
D'Alessandro et al, 2004 [25]	Parents or legal guardians of outpatients and pediatricians	Pediatrics	Mostly White, medically insured, telephone access, English speaking, and some or finished graduate school	197	23-52
Siegel et al, 2006 [9]	T1: physicians and T2: outpatients^b^	Varied	N/A^c^	T1: 270; T2: 907	N/A
Hailemeskel et al, 2007 [26]	Outpatients, student pharmacists, and pharmacy residents	Varied	Predominantly African American women	92	18-35+
Leisey and Shipman, 2007 [27]	Outpatients and physicians	Varied	N/A	110	N/A
Smalligan et al, 2008 [28]	Outpatients and physicians	Varied	Limited internet access among some patients	893	N/A
Zyskind et al, 2009 [29]	Outpatients, physicians, and research assistants	Diabetes	Low socioeconomic status and education, predominantly underserved, and Spanish speaking	108	45-59^d^
McConnaughy and Wilson, 2010 [30]	Parents of children with special needs and librarians	Varied	Varying levels of internet computer literacy	324 visitors and 6 parents	N/A
Coberly et al, 2010 [6]	Outpatients, physicians, staff, and medical librarians	Varied	Majority White, highly educated, secure internet access, and insurance status	224	N/A
Teolis, 2010 [31]	Outpatients, health care professionals, and medical librarians	Varied	Majority unemployed without preventative health care, uninsured, indigent patients, and low health literacy	809	N/A
Ulmer and Robishaw, 2010 [32]	Inpatients, parents, librarians, physicians, and nurses	Varied	Minorities (Spanish-speaking patients) included and health literacy assessed	10	N/A
Lasky et al, 2011 [33]	Outpatients and pharmacy students	Varied	Majority White	198	46-65^d^
Coberly et al, 2012 [7]	Outpatients	Varied	Majority White and well-educated	592	51-52
Gavgani, 2012 [34]	Inpatients, cardiologists, information specialists, and research assistants	Cardiovascular disease	Persians with religion and rural population included	95	34-75^d^
McCarthy et al, 2013 [35]	ED^e^ patients	Varied	Majority non-White, varied education, literacy, health insurance, prescription drug coverage, and computer or internet access	3386	35-54
Tarver et al, 2013 [36]	Outpatients, librarians, and hospital employees	Varied	N/A	5600	N/A
Ramesh et al, 2013 [37]	Participants from Amazon Mechanical Turk	N/A	Majority Asian and White and varied education and health literacy	59	N/A
Borbolla et al, 2014 [4]	Outpatients	Varied	Spanish speaking	N/A	N/A
Koonce et al, 2015 [38]	Outpatients and physicians	Diabetes	Majority African American, high school education or less, household incomes ≤$20,000, employment status, and varied health literacy	160	53-54^d^
Ancker et al, 2016 [5]	Outpatients	Varied	Different genders, races, insurance, and ethnicity, including White, Black, and Latino populations	12,877	45-64
Wilcox et al, 2016 [39]	Inpatients, families, and pharmacists	Cardiothoracic surgery	Sex, age, and varied education levels	32	26-88
Caufield-Noll and Gorman, 2017 [40]	Outpatients, librarians, and physicians	Cardiology	N/A	234	N/A
Sanders et al, 2018 [41]	Outpatients, librarians, clinicians, and nurses	Varied	Minority, low education, and low income	302	Middle aged
Fenske, 2019 [42]	Inpatients, families, nurses, child life specialists, and librarians	Pediatric cancer and chronic illness	N/A	133	N/A
Fawcett, 2020 [43]	Outpatients	Diabetes	Majority college degrees or higher and insurance status	14	N/A
Kazemi Majd et al, 2021 [44]	Inpatients, cardiologists, and librarians	Heart failure	Literate patients or those cared for by a literate person	120	56-80
Zhang et al, 2023 [45]	Individuals from one community	Diabetes and depression	Majority immigrants, low income, low education levels, Spanish as preferred language, and secure internet access	134	18-80+
Yu et al, 2024 [46]	Outpatients	Localized prostate cancer	N/A	10	67-89

a SDOH: social determinants of health.

b T1: first evaluation study; and T2: second evaluation study.

c N/A: not reported in the full-text article.

d The majority of the participants in a study were within the specified age range or means.

e ED: emergency department.

In addition to patient participants, several studies also involved health care professionals: 12 involved clinicians with various backgrounds, 3 targeted nurses, 2 included research assistants, and 2 involved other hospital staff. Community-based participants comprised librarians from medical, health sciences, or public libraries (9 studies) and pharmacists or pharmacy students (3 studies).

SDOH factors were captured in the patients’ demographic data. The studies explicitly addressed underserved and low socioeconomic status groups [29,38,41,45] and participants with low [31] or varied health literacy levels [37,38]. Several studies either focused on or included racial and ethnic populations, such as African American populations [26,38], Spanish-speaking underserved populations [29,32,45], indigent patients [31], Asian populations [37], Persian populations [34], or diverse race populations [5].

Health insurance status was detailed in 6 studies [5,6,25,31,35,43], with 3 indicating that partial participants were uninsured, while 2 reported that the vast majority were insured. While 1 study reported that 148 (75%) of 197 study participants had an annual income exceeding $50,000 [25], other studies reported low income or unemployment among their participants [29,31,38,41,45]. In addition, internet access was addressed in multiple studies. While 3 studies reported secure internet access [6,25,45], 2 studies indicated that participants had limited internet or computer access [28,31], highlighting persistent access barriers relevant to digital health implementation.

The sample sizes in these studies ranged from 10 to 12,877 participants. Most studies had sample sizes under 300 participants, while several studies reported larger samples exceeding 500 participants [5,7,28,31,35,36]. Among studies reporting age, participants ranged from 17 to 88 years, with mean ages typically ranging between 35 and 65 years.

### Intervention

Table 4 presents that 13 studies explicitly identified their main intervention feature as either “information prescription” or “information Rx.” Two of them [6,7] investigated the effectiveness of IPs delivered via paper or email. Additionally, 3 studies [31,33,41] investigated the use of kiosks to deliver multimodal MedlinePlus information. While most studies used MedlinePlus resources on desktops or laptops, 2 studies explored iPad or tablet use [41,42], and 6 studies embedded MedlinePlus information as hyperlinks in patient portals of EHRs [4,5,32,36,37,39].

**Table 4.** Intervention characteristics.

Study	Intervention feature	Information content	Additional interventions	Setting
D'Alessandro et al, 2004 [25]	Information prescription	Three preprinted websites (MedlinePlus, GeneralPediatrics.com, and the American Academy of Pediatrics website)	Hands-on training on internet health information search, handout about computer training, a list of community locations for computer access, and pediatrician training on internet patient education information	Clinical setting
Siegel et al, 2006 [9]	Information Rx	Physicians prescribed specific health information for patients to find on the MedlinePlus	Information Rx kit (pads, posters, and bookmarks) and referral to the MedlinePlus website	Clinical setting
Hailemeskel et al, 2007 [26]	Pharmacy-community partnership	One-on-one medication education and counseling	Student training classes (80 min), MedlinePlus materials, and written instructions	Pharmacy
Leisey and Shipman, 2007 [27]	Information prescription	Physician referral to MedlinePlus	A canvas gift bag containing MedlinePlus and women’s health information	Clinical setting
Smalligan et al, 2008 [28]	Information Rx	Patients' encouragement to use MedlinePlus for a period	N/A^a^	Clinical setting
Zyskind et al, 2009 [29]	Information Rx	MedlinePlus X-plain diabetes tutorials on computer and given Information Rx	N/A	Clinical setting
McConnaughy and Wilson 2010 [30]	Information portal	InfoAble portal with links to health info from MedlinePlus	Other resources (ClinicalTrials.gov, PubMed, and Center for Disability Resource Library)	Library setting
Coberly et al, 2010 [6]	Email information prescription	Information prescription and condition-specific email linking to MedlinePlus topics	Physician and staff training on MedlinePlus by a medical librarian	Clinical setting
Teolis, 2010 [31]	Information Rx kiosk	48 selected MedlinePlus tutorials	MedlinePlus bookmark distribution, built-in printer, and dedicated phone line for librarian assistance	Clinical setting
Ulmer and Robishaw, 2010 [32]	Information Rx via EPIC	MedlinePlus and the library’s subscription-based consumer health databases	Patient health education provided by a nurse	Clinical setting
Lasky et al, 2011 [33]	Pharmacy kiosk	MedlinePlus website	Pharmacist counseling, printed drug labeling and medication guides, and MedlinePlus bookmark and notepads	Pharmacy
Coberly et al, 2012 [7]	Paper versus email information prescription	Email or paper prescription containing 5 links to a study website with specific MedlinePlus web resources	Instructions for accessing a medical librarian for additional information	Clinical setting
Gavgani, 2012 [34]	Information prescription	Culturally adapted MedlinePlus information on coronary artery disease	Information selected from additional sources, translated into Persian, and simplified to grade 6‐7 readability	Clinical setting
McCarthy et al, 2013 [35]	Information prescription	MedlinePlus topics about the prescribed drug	Research assistant-guided review of MedlinePlus drug information, MedlinePlus URL magnet, prescription filling–related information, and medical librarian phone referral	Clinical setting
Tarver et al, 2013 [36]	MedlinePlus resources embedded in patient portals (MyChart)	Contextual information embedded in patient portals via MedlinePlus Connect and MedlinePlus search box	Printed materials, MyChart posters and cards with MedlinePlus resource links, library phone number, and waiting room computer	Public library setting or clinical setting
Ramesh et al, 2013 [37]	NoteAid system linking EHR^b^ notes to patient education materials	EHR links to MedlinePlus content	EHR links to UMLS^c^ and Wikipedia	Patient portal
Borbolla et al, 2014 [4]	Disease-specific information button on home screen and MedlinePlus links embedded in laboratory results screen	Infobutton within a PHR^d^ system linking to MedlinePlus in Spanish	N/A	Patient portal
Koonce et al, 2015 [38]	Information prescription	Diabetes educational materials at 5th- and 8th-grade reading levels, visual, auditory, and kinesthetic formats, Spanish version	N/A	Clinical setting
Ancker et al, 2016 [5]	Hyperlinks containing ICD^e^ codes associated with medical terms	Hyperlinks to MedlinePlus content on the patient portal account	N/A	Patient portal
Wilcox et al, 2016 [39]	Inpatient medication management and tracking	Medication names in patient portals linking to MedlinePlus	Training, demonstration, and observation sessions followed by an application use session with think-aloud protocol	Clinical setting
Caufield-Noll and Gorman 2017 [40]	Patient encounters with library staff for information consultation or instruction	Demonstration of MedlinePlus and AHA^f^ website and informal conversation about a health topic	Patient handout and prepared materials from AHA and NLM^g^	Clinical setting
Sanders et al, 2018 [41]	Computer kiosks and EHR-embedded links	MedlinePlus bookmarks on kiosks and desktop computers in patient examination rooms	Computer classes and MedlinePlus training (group and one-on-one), waiting room demonstrations and recurring MedlinePlus video loops, clinician and nurse training with MedlinePlus integration into after-visit summary reviews, and patient access to iPads and laptops in examination rooms for MedlinePlus access and interactive videos	Clinical setting
Fenske, 2019 [42]	Tablets at bedside	MedlinePlus accessed from tablets	Genetics Home Reference, Genetic and Rare Disease Information Center, X-plain videos, and other NLM resources, nurse training, bookmarks, brochures, and pamphlets produced by NLM or NIH^h^	Clinical setting
Fawcett, 2020 [43]	Multimedia	MedlinePlus video	Educational videos from other resources	Clinical setting
Kazemi Majd et al, 2021 [44]	Information prescription	Physician-prescribed MedlinePlus information and patient-asked additional information	Librarian prepared the health information across multiple sources for patients upon physician prescription	Clinical setting
Zhang et al, 2023 [45]	Dedicated bilingual web portal	Generic MedlinePlus information versus customized MedlinePlus content with localized community components	Community calendar, local service directory, social media integration for content sharing, commenting, and forming groups, Google Translate use, and webpages created by local organizations	Community setting
Yu et al, 2024 [46]	Dedicated web portal	Research team-curated information and MedlinePlus content retrieved via API^i^	Research staff online support available to facilitate prescribed tasks	Online setting via iPICS^j^ platform

a N/A: not reported in the full-text article.

b EHR: electronic health record.

c UMLS: Unified Medical Language System.

d PHR: patient health record.

e ICD: International Classification of Diseases.

f AHA: American Heart Association.

g NLM: National Library of Medicine.

h NIH: National Institutes of Health.

i API: application programming interface.

j iPICS: Interactive prostate cancer information, communication, and support program

Primarily using MedlinePlus web resources, the interventions encompassed a broad range of health information topics, tutorials, and medical term explanations. Additional intervention practices included (1) hands-on computer and health information search training for patients [25,39,41,45]; (2) training for physicians, nurses, staff, and students [6,26,41,42]; (3) the use of other web-based health resources provided by health professionals or organizations [30,34,42,45]; (4) the distribution of supplementary MedlinePlus materials such as bookmarks, pamphlets, brochures, magnets, or waiting room TV loops featuring MedlinePlus videos [9,27,31,33,35,36,40-42]; (5) patient health education or counseling offered by health care professionals as part of the intervention [32,33,35]; and (6) access to librarian assistance, instruction, or training [7,31,35,40,44]. Two recent studies either customized MedlinePlus content with additional local community components (eg, recipes, infographics, and local resource listings) [45] or integrated MedlinePlus into a digital platform with the research team’s self-curated health information [46].

Most studies implemented MedlinePlus interventions in a clinical setting, such as clinics or hospitals, while others used MedlinePlus in community pharmacies [26,33], a community health resource center [45], libraries [30,36], an online platform [46], or patient portals [4,5,37].

### Outcome

Overall, studies reported more nonclinical outcomes than clinical outcomes (Table 5). Of 28 studies, 4 measured clinical outcomes [29,35,43,44]. Two of these studies reported significant differences between the intervention and control groups. For instance, Fawcett [43] reported significant changes in diabetic foot ulcer measurements (*F*_2,26_=3.91; *P*=.03; and *χ*^2^_2_=18.5; *P*<.001). Kazemi Majd et al [44] found fewer patients in the intervention group died or were readmitted compared to the control group during 1 year (relative risk 0.47, 95% CI 0.20‐1.06*; P*=.005), but not in the first 6-month follow-up (relative risk 0.6, 95% CI 0.15‐2.40; *P*=.05). However, the other 2 studies [29,35] reported no significant differences in clinical outcomes between the intervention and control groups, although a difference in patient behavior was noted, with the intervention group being more likely to use the internet or fill their prescriptions.

**Table 5.** Clinical and nonclinical outcomes, comparisons, study duration, and follow-up method.

Study	Clinical outcome	Nonclinical outcome	Comparison	Study duration	Follow-up methods
D'Alessandro et al, 2004 [25]	N/A^a^	Participants’ attitudes and behaviors about using internet health information resources	General internet search: I^b^>C^c^ (*P*=.05); prescribed info search: I>C (*P*<.001); future IP^d^ use intention: I>C (*P*=.02); and recommendation to others: I>C (*P*=.001)	3 mo	Phone survey
Siegel et al, 2006 [9]	N/A	T1^e^: physician attitudes and behavior and patient experience; and T2^f^: perceived impact on patient-physician relationships and perceived health outcome	Majority positive attitudes and experiences, improved referral behavior and medical communication, and better perceived health decisions and health outcomes (no *P* values reported)	T1: 24 mo and T2: 6 mo	T1: mail survey, telephone interview, or focus group; and T2: web survey
Hailemeskel et al, 2007 [26]	N/A	Familiarity with NLM^g^ databases, including MedlinePlus and usage of MedlinePlus	Patients’ familiarity and use of NLM databases and searching for health topics after intervention (*P*<.001); no significant difference in patients’ interest in and confidence using MedlinePlus: (Fisher exact test, *P*=.40); NLM training perceived as beneficial by 91% of respondents	N/A	Phone interview
Leisey and Shipman, 2007 [27]	N/A	Physicians’ unanimous agreement with IP benefits, no patient-reported recognition of the term, and only 14% patient recall of the recommendation	Discrepancy between physician-reported broad IP practice and no patient recognition of the term and between physician-reported encouragement and patient recall	6 mo	Phone interview
Smalligan et al, 2008 [28]	N/A	MedlinePlus usage; competency of accessing; trust, satisfaction, perceived ease to understand; better health decision-making; intent to reuse; and recommendation to others	N/A	N/A	Online or mail survey
Zyskind et al, 2009 [29]	Weight, BMI, blood pressure, HbA_1c_^h^, LDL^i^ cholesterol, blood glucose, and total cholesterol	Patient knowledge about diabetes, verified awareness and use of online resources, including MedlinePlus for health and diabetes	No statistically significant differences between I and C groups for clinical measures; nonclinical measures (I>C): use internet to learn about health (*P*<.003) and diabetes (*P*<.003); and to have heard of MedlinePlus (*P*<.005)	12 mo	Survey
McConnaughy and Wilson, 2010 [30]	N/A	Positive feedback on the InfoAble portal, usability improvement, and portal and engagement tracked via web use statistics	N/A	1 mo	N/A
Coberly et al, 2010 [6]	N/A	Health information–seeking behavior (ie, internet use to find health information, discussion of internet-sourced health information with a provider, heard of MedlinePlus, and MedlinePlus usage) and recall of receiving IP	No significant differences between the I and C groups before the clinical visit in (1) internet use for health information (*P*=.79), (2) discussing internet health information with a provider (*P*=.18), and (3) patient percentage who reported the use of MedlinePlus (15% vs 17%; *P*=.83); the patient percentage who had heard of MedlinePlus (I>C, 35% vs 20%, *P*=.02)	N/A	Mail survey
Teolis, 2010 [31]	N/A	Number of participants who used MedlinePlus for the first time, percentage of health professionals who used MedlinePlus for the first time, and perceived helpfulness and ease of understanding	N/A	8 mo	Ask for experience after using it
Ulmer and Robishaw, 2010 [32]	N/A	(1) Patients’ understanding of condition or treatment, using information to ask physician questions, perceived helpfulness in overall treatment; (2) physicians’ IP use and perceived impact on patients; and (3) the number of librarian-handled prescriptions	N/A	12 mo	Mailing survey for patients and online survey for physicians
Lasky et al, 2011 [33]	N/A	Health information–seeking behavior related to MedlinePlus, intention to return to the website, average encounter duration, and interest in returning to the website	Age-related differences in self-reported prior MedlinePlus usage and interest in returning to the website (*P*<.01)	2 mo	Kiosk prompted feedback soliciting
Coberly et al, 2012 [7]	N/A	Prescription filling rate, website engagement, health information search behavior, perception of information quality, and preference over email versus paper prescription	Higher filling rate for eHIP^j^ patients than pHIP^k^ patients (38% vs 23%; *P*<.001) and eHIP favored for perceived usefulness and ease of use	N/A	Phone and online survey
Gavgani, 2012 [34]	N/A	Total number of patients receiving IP, major topics, drivers, and barriers identification	N/A	4 mo	N/A
McCarthy et al, 2013 [35]	Adverse effects and symptom improvement	Medication adherence, patient satisfaction with prescription drug information, ED^l^ care, and subsequent health care use	Across all 3 sites, no significant differences between usual care and any interventions; at 1 site, subjects receiving practical prescription information or services or a combination of prescription information or service were more likely to fill their prescription than usual care (OR^m^ 1.8, 95% CI 1.0-3.1); no clinically meaningful differences between groups in adverse-effect incidence or subsequent physician visits	>10 mo	Phone interview
Tarver et al, 2013 [36]	N/A	Patient use, interest, and feedback on contextual information links in MyChart	N/A	12 mo	N/A
Ramesh et al, 2013 [37]	N/A	Self-reported EHR^n^ notes comprehension and readability levels	No statistical significance in improving users’ comprehension of EHR notes with MedlinePlus-implemented NoteAid (*P*>.05) and self-reported EHR note comprehension scores ranged between 3 and 4 on a 5-point scale	N/A	N/A
Borbolla et al, 2014 [4]	N/A	Access frequency to MedlinePlus Connect via patient portal and percentage of access sessions on laboratory results	N/A	14 mo	N/A
Koonce et al, 2015 [38]	N/A	Diabetes knowledge scores at 2- and 6-week follow-ups	Diabetes knowledge scores at 2- and 6-week follow-ups: I>C (*P*<.001)	6 mo	Phone interview
Ancker et al, 2016 [5]	N/A	Frequency of patient portal access and usage frequency of MPC^o^	Black patients were more likely to use MPC than White patients (43% vs 40%; *P*<.001); Latino patients were more likely to use the resource than non-Latinos (43% vs 41%; *P*<.001); patients covered by Medicaid were more likely to use MPC than privately insured (44% vs 43%; *P*<.001)	36 mo	N/A
Wilcox et al, 2016 [39]	N/A	Usage frequency, usability, usefulness, acceptability, and audit logs	Perceived usefulness by both pharmacists and patients, preferring different features	19 wk	Interview
Caufield-Noll and Gorman, 2017 [40]	N/A	Patient satisfaction	N/A	21 mo	Survey
Sanders et al, 2018 [41]	N/A	Patients’ frequency of access to MedlinePlus, knowledge, attitude, and skills related to accessing online health resources and clinicians’ use and recommendation of MedlinePlus	Patient use of MedlinePlus increased significantly from 2% before intervention to 6% after intervention (*χ*^2^_1_=5.2; *P*=.02); MedlinePlus recommendation from clinicians and nurses increased from 21% to 43% (*χ*^2^_1_=6.0; *P*<.001)	34 mo	Survey
Fenske, 2019 [42]	N/A	Number of patients and families served, understanding of diagnosis, procedures, and care; generating questions for providers; and confidence in searching MedlinePlus	Improved understanding of diagnosis, procedure, or care after video viewing	10 mo	Survey
Fawcett, 2020 [43]	DFU^p^ area measurement	NAFF scores	No significant change in NAFF^q^ scores after intervention (*t*_13_=−0.28; *P*=.79) and significant change in DFU area (*F*_2,26_=3.91; *P*=.03)	4 wk	NAFF self-evaluation and wound assessment
Kazemi Majd et al, 2021 [44]	Reduction in the hospital readmission and death rate	N/A	One-year mortality: fewer deaths in the intervention group than the control group (7 vs 15; RR^r^ 0.47, 95% CI 0.20‐1.06*; P*=.005); 6-month death or readmission: no statistically significant difference between groups (RR 0.6, 95% CI 0.15‐2.40; *P*=.05)	12 mo	Phone interview
Zhang et al, 2023 [45]	N/A	PAM^s^, disease knowledge, self-efficacy, behavior changes, ease of use and usage of the digital platform, and frequency and methods of using the resource	No statistically significant difference between groups in PAM, disease knowledge, or self-efficacy at 1 month; a significant improvement in PAM scores within intervention group (*P*=.048), but not in the control group (*P*=.06); both groups significantly improved self-efficacy scores on communication with providers, finding professional help, information, and knowledge (*P*<.03)	13 mo	Face-to-face survey
Yu et al, 2024 [46]	N/A	Precision and recall of MedlinePlus search; task completion, time, and number of browsed content types	N/A	1 mo	N/A

a N/A: no relevant information reported in the full-text article.

b I: intervention.

c C: control.

d IP: information prescription.

e T1: first evaluation study.

f T2: second evaluation study.

g NLM: National Library of Medicine.

h HbA1c: hemoglobin A_1c_.

i LDL: low-density lipoprotein.

j eHIP: email health information prescription.

k pHIP: paper health information prescription.

l ED: emergency department.

m OR: odds ratio.

n EHR: electronic health record.

o MPC: MedlinePlus Connect.

p DFU: diabetic foot ulcer area measurement.

q NAFF: Nottingham Assessment of Functional Footcare scores.

r RR: relative risk.

s PAM: Patient Activation Measure.

Most studies (24/28) reported positive nonclinical outcomes, focusing on (1) participants’ attitudes, satisfaction, and behaviors related to online health information resource usage and information-seeking behaviors; (2) frequency and user experience of MedlinePlus usage; and (3) perceived usefulness, trust, ease of use, and competency in using MedlinePlus information for health decision-making, physician communication, self-management, and self-efficacy on finding professional help and disease knowledge.

The study durations varied, typically around 12 months, ranging from 1 to 34 months. Most studies used surveys or interviews for participant follow-ups.

### Quality Assessment

All studies were empirical with clear research goals, objectives, or research questions, and they collected data to address their research questions, achieving MMAT S1 and S2. Among them, 2 qualitative studies were analyzed by MMAT criteria from 1.1 to 1.5, and only one criterion (ie, MMAT 1.1) was met in each study [34,36]. For 7 RCTs evaluated by MMAT 2.1 to 2.5, the number of criteria met ranged from 1 to 5, with many studies lacking sufficient details on either the outcome assessors blinded to the intervention (MMAT 2.4) or follow-up response rates of adherence to the intervention (MMAT 2.5). Each of the 4 nonrandomized studies met at least two criteria (eg, MMAT 3.2 and 3.5), and only one study [5] met MMAT 3.4 by addressing potential confounding variables adequately. Seven quantitative descriptive studies met 2 or 3 criteria (MMAT 4.3 and 4.5), and the majority rarely provided sufficient information about their sampling strategies or reduced nonresponse bias risk (MMAT 4.1, 4.2, and 4.4). Finally, among 8 mixed methods studies, 1 did not meet any criteria, while 6 met only 1 criterion. The study by Wilcox et al [39] met all 5 criteria. Most mixed methods studies lacked sufficient details on whether and how the qualitative and quantitative findings were integrated and interpreted (MMAT 5.3). Overall, when considering the average number of MMAT criteria met per study type, qualitative studies had the fewest met criteria (one per study), while nonrandomized studies had the most (ie, an average of 3.25 criteria per study). Detailed quality assessment results were documented in Multimedia Appendix 2.

## Discussion

### Bibliometrics

As the primary funding agency for half of the included studies, the US NLM has sustained MedlinePlus as a reliable and trusted health information resource. While most included studies took place in the United States, 3 studies were conducted in developing countries, demonstrating MedlinePlus’s applicability and value across cultural, medical, and organizational contexts [4,34,44].

Despite nearly 25 years of availability, MedlinePlus has generated a relatively small body of empirical research examining its role in clinical care and chronic disease management. The limited number of identified studies (n=28), coupled with generally below-average citation impact (ie, FWCI scores <1.0) and below-benchmark Altmetric scores (<20), suggests that MedlinePlus has been underused and underexamined in health care practice and research. These gaps highlighted missed opportunities to rigorously document its implementation effectiveness and potential contribution to patient- and caregiver-centered care.

### Participants

Guided by the CCCM framework, this review highlights the engagement of diverse participant groups with MedlinePlus across coordinated and team-based care settings, including health care professionals, inpatient and outpatient contexts, patients with varied health conditions, family caregivers, pharmacists, and librarians. In particular, medical or public libraries often played a critical role in “physician-directed” information delivery [6], improving community health literacy [47], and serving as community partners to make MedlinePlus accessible to broader audiences. These findings underscore its potential role in integrated care delivery.

Although most studies reported patients’ ages, races, and socioeconomic backgrounds, many lacked SDOH-related specifics such as health insurance, annual income, employment, or health literacy. This gap limits our understanding of how sociodemographics impacted MedlinePlus use, especially given the strong association between health literacy, internet access, and digital engagement [48].

### Intervention

As an information intervention in clinical care, early studies on MedlinePlus [25,26,28] were directly influenced by the paper-based IP or Information Rx program launched by the NLM in 2002. However, later work reflected a growing interest in integrating MedlinePlus into EHR-enabled environments. For example, Ramesh et al [37] highlighted earlier efforts to embed MedlinePlus within Health IT systems. This trend continues, with more recent studies incorporating MedlinePlus into patient portals [4,5,39,41] and stand-alone digital health platforms for patients and caregivers [45,46], supporting their self-management and health information access beyond traditional clinical settings.

Several studies explored the use of kiosks in community pharmacies to deliver multimodal MedlinePlus information [31,33,41], aligning with the community linkage in the CCCM framework. Other studies also reported disseminating MedlinePlus information via iPad or tablet, or combining MedlinePlus content with additional resources, such as the American Heart Association patient materials, RxList, Mayo Clinic, and even religious texts (ie, the Holy Quran). These interventions were implemented across settings, from clinics to virtual environments, highlighting MedlinePlus’ adaptability and versatility in supporting varied models of health information delivery.

### Outcome

Clinical outcomes were reported in only 4 studies. Their results varied from no to significant differences in assessed measures, sometimes with mixed results even within the same study. These inconclusive results indicate the complex nature of directly linking MedlinePlus information interventions to patient health improvements. Researchers identified barriers, including inconsistent physician participation [27], small sample sizes [6], low computer literacy [29] or digital divide [5], limited evaluation periods [29], and information intervention design challenges [35]. Nevertheless, most studies reported nonclinical outcomes, including notable improvements in participants’ attitudes, satisfaction, and behaviors related to online health information seeking. These nonclinical outcomes highlight the benefits of using MedlinePlus in enhancing patients’ health engagement, communication, education, and self-efficacy, providing strong evidence to inform physician-directed health information interventions that complement routine clinical care.

Overall, in light of the identified research gaps, we recommend the following priorities for future research. First, additional studies are needed to rigorously examine the impact of MedlinePlus on patient- and caregiver-centered health outcomes and ensure transparent reporting and effective dissemination of findings within and beyond scholarly communities. Second, more rigorously designed RCTs are needed to evaluate its clinical efficacy. The existing RCTs missed or underreported several key methodological components. Thus, future studies should provide detailed information on allocation concealment, randomization implementation, and follow-up response rates regarding participants’ adherence to the intervention. Third, digital solutions should seamlessly integrate MedlinePlus content into patients’ health care journeys, such as through personalized apps or culturally adapted digital health platforms. Although the most recent 2 studies [45,46] suggest these burgeoning trends, future studies shall ensure that patients receive relevant information tailored to their specific needs, preferences, and literacy levels. Finally, future research shall consistently collect and report SDOH-related demographics, such as income, insurance, education, and health literacy, and support MedlinePlus as an equitable intervention responsive to the care needs of patients and caregivers of different backgrounds.

Despite its global importance, MedlinePlus faces challenges from rapid technological advances (eg, keeping pace with evolving web design and digital infrastructure), competition from commercial products (eg, Healthwise within EPIC EHRs), and uncertainties about the long-term sustainability of federal funding amid federal priority shifts. However, the synthesized evidence from this review demonstrated consistent nonclinical benefits and clinical potentials, validating MedlinePlus’ enduring value and utility. The findings provide evidence to inform future Health IT innovations, investments, and policy decision-making.

### Limitations

This study has several limitations. First, although we aimed to identify all relevant studies, we may have missed those published in languages other than English or not indexed in the 9 bibliographic databases we searched. Second, we excluded articles that reported the use of MedlinePlus for nonpatient or noncaregiver populations. While this exclusion helped us focus on MedlinePlus’s role in clinical care, it might have limited the interpretations of MedlinePlus’s real impact beyond clinical contexts. Third, the included studies are heterogeneous in their research designs, and many lack demographic information on socioeconomic status, constraining a comprehensive understanding of how SDOH-related demographics influence MedlinePlus utilization. Future studies shall address this gap by ensuring more complete and consistent demographic data collection and reporting.

### Conclusions

This systematic literature review is the first to examine how MedlinePlus has been integrated into clinical care, supporting patients and caregivers through increased health information access. Researchers agree that promoting MedlinePlus resources will help address the digital divide, particularly for individuals who are homebound, reside in rural areas, or are underserved. Although health care providers are the key stakeholders in advocating MedlinePlus, the effective delivery and optimization of MedlinePlus health information for patient care requires collective and coordinated community efforts. Our synthesis through the CCCM-guided lens will serve as a foundation for future research and practice in designing and developing accessible, equitable, and scalable digital health information solutions to improve patient engagement, health literacy, self-management, and health outcomes.

## Supplementary material

10.2196/79416 Multimedia Appendix 1 Search strategy.

10.2196/79416 Multimedia Appendix 2 Quality assessment results by Mixed Methods Appraisal Tool.

10.2196/79416 Checklist 1 PRISMA checklist.

## Abbreviations

AAS: Altmetric Attention Score
CCCM: Collaborative Chronic Care Model
EHR: electronic health record
FWCI: field-weighted citation impact
IP: information prescription
IRx: Information Rx
MMAT: Mixed Methods Appraisal Tool
NLM: National Library of Medicine
PICO: problem/population, intervention, comparison, and outcome
PRISMA: Preferred Reporting Items for Systematic Reviews and Meta-Analyses
RCT: randomized controlled trial
SDOH: social determinants of health
