# Supplementary material for: Leveraging MedlinePlus to Improve Health Information Access Among Patients and Caregivers: Systematic Literature Review
Source: JMIR Med Inform. 2026 Apr 27;14:e79416. doi: 10.2196/79416 (PMC13119385; doi:10.2196/79416)
Supplement: Multimedia Appendix 1 [file medinform-v14-e79416-s001.docx]

### Appendix A: Search Strategy and Results

| Databases | Search queries | Oct. 26, 2021 | June 16, 2022 Update | Sept. 28, 2024 Update |
| --- | --- | --- | --- | --- |
| PubMed | ("MedlinePlus") OR ("Medline Plus") [All Fields] | 230 | 9 | 45 |
| Scopus | ( TITLE-ABS-KEY ( "MedlinePlus" )  OR  TITLE-ABS-KEY ( "Medline Plus" ) ) | 356 | 19 | 29 |
| WoS | "MedlinePlus" OR "Medline Plus"(All Fields) | 234 | No access due to institutional subscription cancellation | No access due to institutional subscription cancellation |
| Embase | ‘medlineplus’:ab,ti OR ‘medline plus’:ab,ti | 254 | 27 | 25 |
| CINAHL via EBSCO | MedlinePlus OR “Medline Plus”[ALL] | 254 | 5 | 0 |
| Global Health via EBSCO | MedlinePlus OR “Medline Plus”[ALL] | 62 | 2 | 0 |
| PsycInfo via  EBSCO | MedlinePlus OR “Medline Plus”[ALL] | 31 | 1 | 0 |
| Cochrane Library | MedlinePlus OR (“Medline Plus”)[Title Abstract Keyword] | 12 | 0 | 0 |
| Dimensions | MedlinePlus OR (“Medline Plus”) Search in: Title and Abstract | No access | No access | 365 |
