# Supplementary material for: Leveraging MedlinePlus to Improve Health Information Access Among Patients and Caregivers: Systematic Literature Review
Source: JMIR Med Inform. 2026 Apr 27;14:e79416. doi: 10.2196/79416 (PMC13119385; doi:10.2196/79416)
Supplement: Multimedia Appendix 2 [file medinform-v14-e79416-s002.docx]

### Appendix B: Quality Assessment Results by Mixed Methods Appraisal Tool (MMAT)

| Study | Category of Study Designs | MMAT Criteria Assessment | | | |
| --- | --- | --- | --- | --- | --- |
|  |  | Criteria | Yes | No | Can’t Tell |
| D'Alessandro 2004 [[25]](https://sciwheel.com/work/citation?ids=4944381&pre=&suf=&sa=0&dbf=0) | RCT | 2.1 |  | x |  |
|  |  | 2.2 | ✓ |  | x |
|  |  | 2.3 |  | x |  |
|  |  | 2.4 |  | x |  |
|  |  | 2.5 |  | x |  |
| Siegel 2006 [[9]](https://sciwheel.com/work/citation?ids=1435876&pre=&suf=&sa=0&dbf=0) | T1: Mixed Methods | 5.1 |  | x |  |
|  |  | 5.2 |  | x |  |
|  |  | 5.3 |  | x |  |
|  |  | 5.4 |  |  | x |
|  |  | 5.5 |  | x |  |
|  | T2: Quantitative Descriptive Study | 4.1 |  | x |  |
|  |  | 4.2 |  | x |  |
|  |  | 4.3 | ✓ |  |  |
|  |  | 4.4 |  |  | x |
|  |  | 4.5 | ✓ |  |  |
| Hailemeskel 2007 [[26]](https://sciwheel.com/work/citation?ids=12015554&pre=&suf=&sa=0&dbf=0) | Quantitative Descriptive Study | 4.1 |  | x |  |
|  |  | 4.2 |  |  | x |
|  |  | 4.3 | ✓ |  |  |
|  |  | 4.4 |  |  | x |
|  |  | 4.5 | ✓ |  |  |
| Leisey 2007[[27]](https://sciwheel.com/work/citation?ids=14689413&pre=&suf=&sa=0&dbf=0) | Mixed Methods | 5.1 |  |  | x |
|  |  | 5.2 | ✓ |  |  |
|  |  | 5.3 |  |  | x |
|  |  | 5.4 |  |  | x |
|  |  | 5.5 |  |  | x |
| Smalligan 2008 [[28]](https://sciwheel.com/work/citation?ids=6263731&pre=&suf=&sa=0&dbf=0) | Quantitative Descriptive Study | 4.1 |  | x |  |
|  |  | 4.2 |  | x |  |
|  |  | 4.3 | ✓ |  |  |
|  |  | 4.4 |  |  | x |
|  |  | 4.5 | ✓ |  |  |
| Zyskind 2009 [[29]](https://sciwheel.com/work/citation?ids=12015595&pre=&suf=&sa=0&dbf=0) | RCT | 2.1 |  |  | x |
|  |  | 2.2 | ✓ |  |  |
|  |  | 2.3 |  | x |  |
|  |  | 2.4 |  | x |  |
|  |  | 2.5 |  |  | x |
| McConnaughy 2010 [[30]](https://sciwheel.com/work/citation?ids=12015572&pre=&suf=&sa=0&dbf=0) | Mixed Methods | 5.1 | ✓ |  |  |
|  |  | 5.2 |  |  | x |
|  |  | 5.3 |  |  | x |
|  |  | 5.4 |  |  | x |
|  |  | 5.5 |  |  | x |
| Coberly 2010 [[6]](https://sciwheel.com/work/citation?ids=11028696&pre=&suf=&sa=0&dbf=0) | Non-randomized Studies | 3.1 |  |  | x |
|  |  | 3.2 | ✓ |  |  |
|  |  | 3.3 |  |  | x |
|  |  | 3.4 |  | x |  |
|  |  | 3.5 | ✓ |  |  |
| Teolis 2010 [[31]](https://sciwheel.com/work/citation?ids=12015565&pre=&suf=&sa=0&dbf=0) | Mixed Methods | 5.1 | ✓ |  |  |
|  |  | 5.2 |  |  | x |
|  |  | 5.3 |  |  | x |
|  |  | 5.4 |  |  | x |
|  |  | 5.5 |  |  | x |
| Ulmer 2010 [[32]](https://sciwheel.com/work/citation?ids=4691540&pre=&suf=&sa=0&dbf=0) | Mixed Methods | 5.1 | ✓ |  |  |
|  |  | 5.2 |  |  | x |
|  |  | 5.3 |  |  | x |
|  |  | 5.4 |  |  | x |
|  |  | 5.5 |  | x |  |
| Lasky 2011 [[33]](https://sciwheel.com/work/citation?ids=12015570&pre=&suf=&sa=0&dbf=0) | Mixed Methods | 5.1 | ✓ |  |  |
|  |  | 5.2 |  | x |  |
|  |  | 5.3 |  |  | x |
|  |  | 5.4 |  |  | x |
|  |  | 5.5 |  |  | x |
| Coberly 2012 [[7]](https://sciwheel.com/work/citation?ids=12015531&pre=&suf=&sa=0&dbf=0) | RCT | 2.1 | ✓ |  |  |
|  |  | 2.2 | ✓ |  |  |
|  |  | 2.3 |  | x |  |
|  |  | 2.4 |  | x |  |
|  |  | 2.5 |  |  | x |
| Gavgani 2012 [[34]](https://sciwheel.com/work/citation?ids=14602282&pre=&suf=&sa=0&dbf=0) | Qualitative Studies | 1.1 | ✓ |  |  |
|  |  | 1.2 |  |  | x |
|  |  | 1.3 |  |  | x |
|  |  | 1.4 |  |  | x |
|  |  | 1.5 |  |  | x |
| McCarthy 2013 [[35]](https://sciwheel.com/work/citation?ids=12015532&pre=&suf=&sa=0&dbf=0) | RCT | 2.1 | ✓ |  |  |
|  |  | 2.2 | ✓ |  |  |
|  |  | 2.3 |  | x |  |
|  |  | 2.4 |  | x |  |
|  |  | 2.5 |  |  | x |
| Tarver 2013 [[36]](https://sciwheel.com/work/citation?ids=634997&pre=&suf=&sa=0&dbf=0) | Qualitative Studies | 1.1 | ✓ |  |  |
|  |  | 1.2 |  |  | x |
|  |  | 1.3 |  |  | x |
|  |  | 1.4 |  |  | x |
|  |  | 1.5 |  |  | x |
| Ramesh 2013 [[37]](https://sciwheel.com/work/citation?ids=12015547&pre=&suf=&sa=0&dbf=0) | Non-randomized studies | 3.1 |  |  | x |
|  |  | 3.2 | ✓ |  |  |
|  |  | 3.3 | ✓ |  |  |
|  |  | 3.4 |  |  | x |
|  |  | 3.5 | ✓ |  |  |
| Borbolla 2014 [[4]](https://sciwheel.com/work/citation?ids=9441386&pre=&suf=&sa=0&dbf=0) | Quantitative Descriptive Studies | 4.1 |  |  | x |
|  |  | 4.2 |  |  | x |
|  |  | 4.3 | ✓ |  |  |
|  |  | 4.4 |  |  | x |
|  |  | 4.5 | ✓ |  |  |
| Koonce 2015 [[38]](https://sciwheel.com/work/citation?ids=4409713&pre=&suf=&sa=0&dbf=0) | RCT | 2.1 | ✓ |  |  |
|  |  | 2.2 | ✓ |  |  |
|  |  | 2.3 |  | x |  |
|  |  | 2.4 |  |  | x |
|  |  | 2.5 |  | x |  |
| Ancker 2016 [[5]](https://sciwheel.com/work/citation?ids=9441305&pre=&suf=&sa=0&dbf=0) | Non-randomized Studies | 3.1 | ✓ |  |  |
|  |  | 3.2 | ✓ |  |  |
|  |  | 3.3 | ✓ |  |  |
|  |  | 3.4 | ✓ |  |  |
|  |  | 3.5 | ✓ |  |  |
| Wilcox 2016 [[39]](https://sciwheel.com/work/citation?ids=5953310&pre=&suf=&sa=0&dbf=0) | Mixed Methods | 5.1 | ✓ |  |  |
|  |  | 5.2 | ✓ |  |  |
|  |  | 5.3 | ✓ |  |  |
|  |  | 5.4 | ✓ |  |  |
|  |  | 5.5 | ✓ |  |  |
| Caufield-Noll 2017 [[40]](https://sciwheel.com/work/citation?ids=4251111&pre=&suf=&sa=0&dbf=0) | Quantitative Descriptive Studies | 4.1 |  |  | x |
|  |  | 4.2 |  |  | x |
|  |  | 4.3 | ✓ |  |  |
|  |  | 4.4 |  | x |  |
|  |  | 4.5 | ✓ |  |  |
| Sanders 2018 [[41]](https://sciwheel.com/work/citation?ids=9441232&pre=&suf=&sa=0&dbf=0) | Mixed Methods | 5.1 | ✓ |  |  |
|  |  | 5.2 |  | x |  |
|  |  | 5.3 |  | x |  |
|  |  | 5.4 |  | x |  |
|  |  | 5.5 | ✓ |  |  |
| Fenske 2019[[42]](https://sciwheel.com/work/citation?ids=9438690&pre=&suf=&sa=0&dbf=0) | Quantitative Descriptive Studies | 4.1 | ✓ |  |  |
|  |  | 4.2 |  | x |  |
|  |  | 4.3 | ✓ |  |  |
|  |  | 4.4 |  | x |  |
|  |  | 4.5 | ✓ |  |  |
| Fawcett 2021[[43]](https://sciwheel.com/work/citation?ids=12015577&pre=&suf=&sa=0&dbf=0) | Non-randomized Studies | 3.1 | ✓ |  |  |
|  |  | 3.2 | ✓ |  |  |
|  |  | 3.3 |  | x |  |
|  |  | 3.4 |  | x |  |
|  |  | 3.5 | ✓ |  |  |
| Kazemi Majd 2021[[44]](https://sciwheel.com/work/citation?ids=11356932&pre=&suf=&sa=0&dbf=0) | RCT | 2.1 | ✓ |  |  |
|  |  | 2.2 | ✓ |  |  |
|  |  | 2.3 | ✓ |  |  |
|  |  | 2.4 | ✓ |  |  |
|  |  | 2.5 | ✓ |  |  |
| Zhang 2023 [[45]](https://sciwheel.com/work/citation?ids=17195744&pre=&suf=&sa=0&dbf=0) | RCT | 2.1 |  |  | x |
|  |  | 2.2 | ✓ |  |  |
|  |  | 2.3 |  | x |  |
|  |  | 2.4 |  | x |  |
|  |  | 2.5 |  |  | x |
| Yu 2024 [[46]](https://sciwheel.com/work/citation?ids=16941409&pre=&suf=&sa=0&dbf=0) | Quantitative Descriptive Studies | 4.1 | ✓ |  |  |
|  |  | 4.2 |  | x |  |
|  |  | 4.3 | ✓ |  |  |
|  |  | 4.4 |  | x |  |
|  |  | 4.5 | ✓ |  |  |

^a^RCT = Randomized Controlled Trial
